# The effects of resistance exercise on cognitive function, amyloidogenesis, and neuroinflammation in Alzheimer’s disease

**DOI:** 10.3389/fnins.2023.1131214

**Published:** 2023-03-02

**Authors:** Caroline Vieira Azevedo, Debora Hashiguchi, Henrique Correia Campos, Emilly V. Figueiredo, Sthefanie Ferreira S. D. Otaviano, Arlete Rita Penitente, Ricardo Mario Arida, Beatriz Monteiro Longo

**Affiliations:** ^1^Department of Physiology, Universidade Federal de São Paulo, São Paulo, Brazil; ^2^Instituto do Cérebro, Universidade Federal do Rio Grande do Norte (UFRN), Natal, Brazil; ^3^Escola de Medicina, Departamento de Ginecologia Obstetrícia e Propedêutica da, Universidade Federal de Ouro Preto (UFOP), Minas Gerais, Brazil

**Keywords:** Alzheimer’s disease, resistant physical exercise, neuroprotection, animal models of AD, patients with AD

## Abstract

With the increasing prevalence of Alzheimer’s disease (AD) and difficulties in finding effective treatments, it is essential to discover alternative therapies through new approaches. In this regard, non-pharmacological therapies, such as physical exercise, have been proposed and explored for the treatment of AD. Recent studies have suggested that resistance exercise (RE) is an effective strategy for promoting benefits in memory and cognitive function, producing neuroprotective and anti-inflammatory effects, and reducing amyloid load and plaques, thereby reducing the risk, and alleviating the neurodegeneration process of AD and other types of dementia in the elderly. In addition, RE is the exercise recommended by the World Health Organization for the elderly due to its benefits in improving muscle strength and balance, and increasing autonomy and functional capacity, favoring improvements in the quality of life of the elderly population, who is more likely to develop AD and other types of dementia. In this mini-review, we discuss the impact of RE on humans affected by MCI and AD, and animal models of AD, and summarize the main findings regarding the effects of RE program on memory and cognitive functions, neurotrophic factors, Aβ deposition and plaque formation, as well as on neuroinflammation. Overall, the present review provides clinical and preclinical evidence that RE plays a role in alleviating AD symptoms and may help to understand the therapeutic potential of RE, thereby continuing the advances in AD therapies.

## 1. Introduction

Alzheimer’s disease (AD) is a neurodegenerative disorder clinically characterized by progressive deficit in cognitive function and emotional behavior, mainly resulting from selective neuronal dysfunction, synaptic loss and neuroinflammation (Hebert et al., 1995). The extensive deposition of amyloid-β peptide (Aβ) in the form of senile plaques in the cortex and hippocampus and the presence of neurofibrillary tangles are the main pathophysiological characteristics of AD. In addition, AD is strongly associated with inflammatory processes resulting in neurotoxicity (Citron, 2010; Duncan and Valenzuela, 2017). With the exponential growth of the elderly population, AD presents a broad spectrum of neuropathological changes and associated risk factors. Demographic projections suggest that the disease accounts for 60-70% of dementia cases worldwide, and with increasing life expectancy, the number of AD cases has gradually increased, with an estimated 150 million cases by 2050 (Rosenberg et al., 2020; World Health Organization [WHO], 2022). Despite great advances in AD research, significant efficacy in the treatment or prevention of the disease has not yet been achieved.

Loss of strength and muscle atrophy (sarcopenia) occur frequently in the elderly and individuals with AD (Beeri et al., 2021). The consequences of this loss of function and muscle sarcopenia lead to greater difficulty in being physically active, and interfere with quality of life, thus being a major concern for the clinical population with AD and other types of dementia, such as mild cognitive impairment (MCI) (Beeri et al., 2021).

Recent studies have consistently demonstrated the beneficial effects of physical exercise on the neuropathology of AD (Kametani and Hasegawa, 2018). Among several proposed physical interventions, resistance exercise (RE) is the recommended exercise for older people, according to the World Health Organization (Bull et al., 2020). RE is characterized by contractions of specific muscles against external resistance, and has emerged as an essential strategy to improve muscle mass and strength, bone density, and power of the overall body composition, as well as functional capacity and balance, thereby attenuating or even reversing sarcopenia and reducing difficulties in task performance (Lopez et al., 2018; Smith et al., 2022).

**FIGURE 1:**
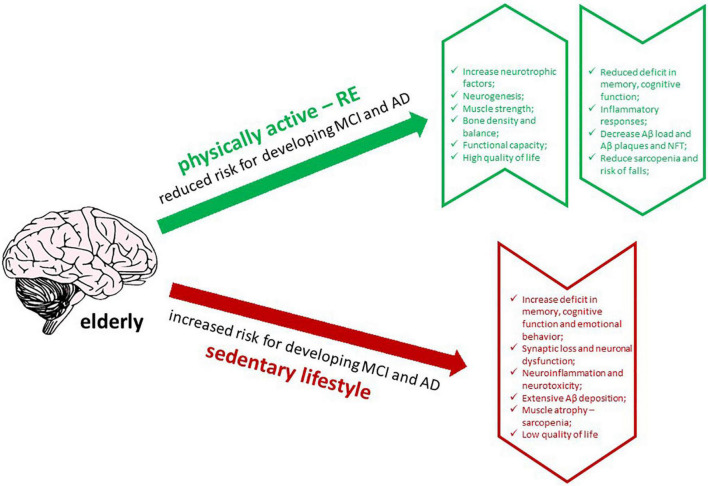
Graphic abstract of the effects of RE in AD brain (created with BioRender.com).

In addition to its physical benefits, RE has been proposed to improve cognitive function and memory in the elderly and AD patients (Ozkaya et al., 2005; Cassilhas et al., 2007; Liu-Ambrose et al., 2012; Almeida et al., 2022). Moreover, RE produces neuroprotective effects, thereby increasing the release of neurotrophic factors, promoting neurogenesis, modulating inflammatory responses, and reducing Aβ load in humans with AD (Navarro et al., 2018; Almeida et al., 2022). Thus, insights into the effects of RE on AD will be helpful in understanding how it could interfere with the affected brain, regarding its effects on memory and cognitive functions, neurotrophic factors, Aβ deposition and plaque formation, and neuroinflammation. In this review, we discuss the impact of RE and its ability to alleviate the pathogenesis of AD in humans affected by MCI and AD, and in experimental animal models of AD, focusing on the main studies that have addressed these issues (Figure 1).

### 1.1. Effects of RE on cognitive function and memory in AD

Interest in RE as a strategy for treating or preventing MCI and AD has grown since recent studies have shown its effectiveness in treating cognitive function and memory in patients with MCI or AD (Table 1; Vital et al., 2012; Fiatarone Singh et al., 2014; Brinke et al., 2015; Hong et al., 2017; Mavros et al., 2017; Tsai et al., 2018; Broadhouse et al., 2020). Among them, the study by Hong et al. (2017) showed that RE recovered cognitive function in elderly patients with MCI. This study demonstrated the effectiveness of RE in these patients, proving that RE frequency, intensity, and duration are important factors to consider. Moreover, six months of RE significantly improved memory performance, attention, and executive functions (Fiatarone Singh et al., 2014; Mavros et al., 2017), and protected the hippocampus from the degeneration that occurs in AD (Broadhouse et al., 2020). These benefits persisted for 12 months after the end of the training period (Fiatarone Singh et al., 2014; Broadhouse et al., 2020). These findings emphasize the therapeutic potential of RE for the prevention and treatment of MCI and AD.

**TABLE 1:** Summary of studies using RE in patients with MCI or AD and in animal models of AD.

RE studies in MCI or AD patients								
Age (years)	Health status	Sample size/ Group	Intervention duration	Intensity	Frequency/ Week	Series/ Exercise	RE short term changes	References
69.5 ± 6.6	MCI	13-22	6 months	High (80% of peak capacity)	2/3	3x 8 of 5/6 exercises	↑ Cortical and hippocampal thickness	Broadhouse et al., 2020
≥ 55	MCI	17-27	6 months	High (80-92% of 1 maximum repetitions)	2/3	3x 8 of 5/6 exercises	↑ Body strenght Cognitive improvement	Mavros et al., 2017
≥ 55	MCI	22-27	6 months	High (↑)	2/3	3x 8 of 5/6 exercises	Mixed memory outcomes Cognitive and executive improvement	Fiatarone Singh et al., 2014
70-80 women	MCI	08-27	6 months	13-15 Borg’s RPE scale	2	2x 6-8 of 7 exercises	No differerence in hippocampal volume	Brinke et al., 2015
75.53 ± 4.48	MCI	10-13	12 weeks	65% of 1RM	2	15 maximum repetitions no info about exercises	↑ Physical fitness ↑ Body muscular strengh No effect in general cognition but working memory improvement	Hong et al., 2017
Not informed	AD	17	16 weeks	85% of 20 maximum repetitions	3	2x 20 + 1x until fatigue	No difference in cognition and memory	Vital et al., 2012
60-80	MCI	20-25	1 day	75% of 1 maximal repetition	1	2x 10 of 6 exercises	↑ serum IGF-1	Tsai et al., 2018
**RE studies in AD animal models**								
**Animal model**	**Age (months)**	**Sample size/** **Group**	**Intervention duration**	**Intensity**	**Frequency/** **Week**	**Repetitions**	**RE changes**	
Female D-GAL (Wistar)	6-7	8	6 weeks	Load increasing from 50-100% of body mass	3	8	Muscle hypertrophy Cognitive improvement ↓ hippocampal and cortex Aβ No difference in hippocampal BDNF ↑ serum IGF-1 (not in the brain)	Özbeyli et al., 2017
Male APP/PS1 (C57Bl/6)	6-7	05-15	4 weeks	Load increasing from 75-100% of maximal load	5	2x each % body mass + 100% +3g until falied	↓ hyperlocomotion ↓ hippocampal Aβ plaques volume ↓ hippocampal microglia around Aβ plaques ↓ hippocampal pro-inflammatory IL-1α, IL-6 cytokines ↓ hippocampal anti-inflammatory IL-4 cytokine	Hashiguchi et al., 2020
Male treated with STZ (Wistar)	Adults	Not informed	8 weeks	Load increasing from 30-150% of body mass	3	Not informed	Memory improvement ↑ hippocampal BDNF and NGF and NT3 ↑ hippocampal TrkA and TrkB	Jafarzadeh et al., 2021
Male 3xTg-AD (mice)	3	10	9 weeks	Load increasing from 50- 100% of body mass	3	16-10	↑ gastrocnemius mass ↑ grip strenght ↓ hippocampus Aβ load No difference in BDNF ↑ IGF-1	Pena et al., 2020
Male 3xTg Psen (B6) mice	9	07-08	4 weeks	Load increasing from 15- 75% of body mass	3	15	Cognitive improvement ↓ frontal cortex and hippocampal Aβ plaques ↓ frontal cortex total and phosphorilated tau ↓ hippocampal phosphorilated tau ↓ frontal cortex and hippocampal micróglia ↓ frontal cortex and hippocampal microglia cell body ↑ hippocampal anti-inflammatory IL-10 cytokine ↓ serum IL-1β cytokine ↑ frontal cortex and hippocampal pro-inflammatory IL-6 cytokine	Liu et al., 2020

Studies in animal models of AD have also shown the positive effects of RE on memory deficits and cognitive dysfunction (Table 1; Özbeyli et al., 2017; Liu et al., 2020; Pena et al., 2020; Jafarzadeh et al., 2021). Liu et al. found that RE could improve cognitive function in AD mice by reducing amyloid load and tau pathology, and attenuating neuroinflammation. These authors proposed that one of the mechanisms by which RE promotes these beneficial effects is an increase in pre-synaptic structural proteins involved in recycling vesicles consequently improving synaptic transmission (Liu et al., 2020). In another study, Pena et al. found that RE induced mechanisms of neuronal protection and reduction of Aβ load in the hippocampus, which in turn could improve learning and memory processing (Pena et al., 2020). Özbeyli et al. (2017) showed that the improvement in working memory provided by RE may occur via oxidative stress and the antioxidant system. Another study by Jafarzadeh et al. (2021) used a model of intraventricular injection of streptozotocin (STZ) in rats and observed that RE improved learning and memory, together with an increase in the expression of neurotrophic factors and their respective receptors. Although by different mechanisms, these studies showed improvements in memory and cognitive deficits in AD experimental models, and corroborate the results from studies with MCI patients.

Interestingly, these and other studies have shown that RE combined with aerobic exercise yields good results in improving memory and cognitive functions and minimizing AD pathophysiology (Hong et al., 2017; Özbeyli et al., 2017; De la Rosa et al., 2020; Pena et al., 2020; Huuha et al., 2022). Although only few studies have investigated the effects of RE on memory deficits presented in MCI and AD (Table 1), conventional aerobic exercise protocols have been extensively explored to investigate this issue (Sousa et al., 2021; Almeida et al., 2022). Considerable individual differences related to the degree and severity of AD pathology in memory and cognitive performance may be partially explained by the concept of cognitive reserve, which describes the brain’s functional ability to adapt and compensate for damage. Furthermore, considering that memory improvement is related to the formation of new hippocampal neurons, as shown in physically active AD animals, studies have proposed that exercise-induced neurogenesis may improve memory and help recovering from cognitive deficits that occur in AD (Mirochnic et al., 2009; Tapia-Rojas et al., 2016; Gaitán et al., 2021). Thus, increased cognitive reserve may help explain the reduced risk of AD in exercised patients and animals (Huuha et al., 2022). Therefore, it is plausible that the combination of different exercise modalities is more efficient in increasing cognitive reserve and neurogenesis than exercise programs with a single type of exercise.

### 1.2. Influence of RE on neurotrophic factors in AD

The direct action of brain-derived neurotrophic factor (BDNF) and nerve growth factor (NGF) on neuronal survival and plasticity has led to the investigation of these neurotrophins in AD, as neuronal death and synaptic loss are among the main neuropathological features of the disease. It has been shown that in neuronal culture, the availability of BDNF and NGF increases the viability of cells previously exposed to the Aβ peptide. Conversely, a decrease in the availability of BDNF and TrkA receptors, which impairs NGF signaling, increases Aβ and Tau levels. These studies suggest that BDNF and NGF are both involved in amyloid precursor protein (APP) processing (for review see Gao et al., 2022). *In vivo* studies corroborate these findings and provide evidence for the benefits of neurotrophic factors in neuronal survival. While low levels of BDNF are associated with cognitive deficits and impaired memory and learning (Capsoni et al., 2000; Heldt et al., 2007), increasing BDNF levels in AD animals prevents neuronal loss and improves cognitive performance (Nagahara et al., 2009, 2013; Jiao et al., 2016; Gao et al., 2022). Despite these positive results, other studies have not systematically shown direct interference of BDNF and NGF in the formation of Aβ plaques and neurofibrillary tangles in the development of AD (Capsoni et al., 2000; Arancibia et al., 2008; Castello et al., 2012; Xiong et al., 2015; Braun et al., 2017; Wang et al., 2019), which can be explained by the differences in animal models or methodologies employed.

RE applied in old animals or animal models of AD, using exercise programs lasting between 4 and 12 weeks, increased BDNF levels in the hippocampus (Özbeyli et al., 2017; Vilela et al., 2017; Jafarzadeh et al., 2021; Kim et al., 2021; Lee et al., 2022; Rahmati et al., 2022). Two of these studies also showed increased levels of exercise-induced NGF (Jafarzadeh et al., 2021; Rahmati et al., 2022), whereas Pena et al. found no difference in BDNF levels in the brain of resistance-trained animals (Pena et al., 2020). Human studies investigating the effects of RE in the elderly population have indicated that programs with 12 weeks to 6 months of exercise increase peripheral BDNF levels (Roh et al., 2020; Deus et al., 2021; Castaño et al., 2022). Furthermore, higher BDNF levels were observed after a single RE session (Arazi et al., 2021).

In addition to BDNF and NGF, insulin-like growth factor 1 (IGF-1) is also down-regulated in AD. It has been shown that IGF-1 plays a role in the clearance of Aβ plaques in the cortex and hippocampus (Carro et al., 2002; Talbot et al., 2012). Furthermore, cerebral uptake of IGF-1 increased BDNF mRNA expression (Carro et al., 2002). These studies suggest an inverse correlation between IGF-1 levels and Aβ load and a direct correlation between IGF-1 and BDNF levels in the brain.

Regular physical exercise stimulates synthesis and increases IGF-1 levels, mediates hippocampal neurogenesis, and induces neuroprotection (Carro et al., 2002; Cassilhas et al., 2007; Chang et al., 2012). Importantly, the low IGF-1 levels present in the brain of patients with MCI can be elevated with the practice of RE, helping to increase synaptic plasticity, neuronal survival, and cognitive performance, consequently improving AD pathophysiology (Tsai et al., 2018; Ribariè, 2022). Furthermore, RE elevated the levels of IGF-1 and BDNF by enhancing muscle strength (Burns et al., 2010; Mavros et al., 2017), increasing neurogenesis, and ameliorating insulin sensitization (Kim and Song, 2018; Lourenco et al., 2020). Studies using animal models of AD have also shown that RE helps to increase IGF-1 signaling and reduce APP mRNA and Aβ levels. (Özbeyli et al., 2017; Pena et al., 2020). These results suggest that RE affects the modulation of neurotrophic factors, which in turn plays a protective role in AD pathology, reducing the toxicity resulting from Aβ load in the brain.

When both types of exercise were compared, studies have suggested that RE elevates the levels of IGF-1 more than BDNF in the hippocampus and peripheral blood, whereas aerobic exercise preferentially increase BDNF more than IGF-1 (Cassilhas et al., 2012, Tsai et al., 2018). However, in the study by Pena et al. with 3X-tgAD mice subjected to aerobic exercise and RE, although a concomitant reduction in Aβ load with an increase in IGF-1 levels was observed in the RE group, no significant change was observed in hippocampal BDNF levels under either exercise condition (aerobic or RE). Interestingly, in human studies, serum concentrations of BDNF and VEGF in elderly individuals with MCI increased after acute aerobic exercise but not after acute RE (Tsai et al., 2018). Although the potential mechanisms of the benefits of different types of exercise are not yet clear (Balbim et al., 2022), studies have proposed that RE is likely to exert its effects via mechanisms distinct from those of aerobic exercise (Tsai et al., 2018).

### 1.3. Effects of RE on amyloid load and Aβ plaques in the AD brain

Although the pathological mechanism of AD is not yet well understood, the theory of the Aβ protein as a triggering agent is well accepted. In recent decades, several studies have indicated that the accumulation of Aβ can precede the clinical symptoms of the disease by years, in addition to triggering exacerbated inflammatory processes and accelerating tau pathology. In addition, the intracellular increase in oligomeric forms of Aβ preceding the formation of extracellular Aβ plaques has been proposed as a key early event in AD progression (for review see Huuha et al., 2022).

Evidence consistently indicates an inverse correlation between exercise and amyloid load in AD animals, regardless of the type, intensity, and duration of exercise, and stage of the disease (Zhang J. et al., 2018; Brown et al., 2019; Almeida et al., 2022). Human studies also show an inverse correlation between plasma and brain levels of Aβ and physical activity in the elderly with AD or MCI (for review see De la Rosa et al., 2020), although few studies have found no evidence that exercise reduces Aβ or relieves amyloid pathology (Huuha et al., 2022).

In animal models, RE was able to promote the clearance of Aβ (Özbeyli et al., 2017; Liu et al., 2020; Pena et al., 2020), reduce the volume (Hashiguchi et al., 2020), and number of Aβ plaques (Liu et al., 2020), and reduce tau pathology in the brain (Liu et al., 2020). Accordingly, in the study by Pena et al. there was a reduction in the expression of Aβ peptide, although the levels of the protein APP did not decrease in the hippocampus of 3xTg-AD mice exercised by RE (Pena et al., 2020).

Several mechanisms by which exercise alleviates amyloid pathology have been proposed, including the downregulation of the enzymes responsible for the formation of Aβ (Alkadhi and Dao, 2018; Zhang X. et al., 2018), modulation of microglial activity (Hashiguchi et al., 2020; Liu et al., 2020), and reduction of astrogliosis (Liu et al., 2020). However, as previously suggested, Aβ levels are resistant to clearance once they reach a severity threshold with age (Intlekofer and Cotman, 2013), and exercise may have a greater effect on amyloid pathology in the early stages of the disease (Huuha et al., 2022). Moreover, the benefits of exercise in modulating the burden of Aβ in the pathology of AD may be dependent on the type of exercise, and the stage of the disease, as they can promote similar benefits through different mechanisms and response windows to induce a modifier effect on Aβ (Hashiguchi et al., 2020).

In general, as in RE, it has also been shown that aerobic exercise contributes to the reduction of Aβ load, and size and quantity of Aβ plaques, which consequently improves memory (Tapia-Rojas et al., 2016; Lu et al., 2017; Choi et al., 2018). In contrast, some studies on aerobic exercise in animal models of AD, as in humans, did not find a decrease in the Aβ plaques (Ke et al., 2011), or reduced only in younger but not in older mice (Zhao et al., 2015). Accordingly, although physical exercise induced positive effects on synapse strength, redox homeostasis, and general brain function, it was not able to reduce the hippocampal levels of Aβ deposition (García-Mesa et al., 2011). Interestingly, when comparing both aerobic and RE training, Pena et al. observed a reduction in Aβ levels in RE but not in aerobic exercised AD mice, which suggests that it would be reasonable to combine RE with other types of physical exercise as a more effective therapeutic strategy in reducing Aβ.

### 1.4. RE modulating inflammatory responses in AD

An exacerbated inflammatory response in the brain of patients with cognitive dysfunction has been observed, and has been identified as a risk factor for neurodegenerative diseases (Intlekofer and Cotman, 2013). In AD, elevated levels of pro-inflammatory cytokines, such as IL-1β, IL-6, IL-12, IL-18, TNF-α, and IFNγ, have been observed in both patients and AD experimental models in rodents (Alvarez et al., 2007; Bronzuoli et al., 2016; Wu et al., 2018; Jensen et al., 2019; Hashiguchi et al., 2020; Li et al., 2020; Liu et al., 2020). Additionally, a lower ability to clear Aβ plaques is associated with an increase in the levels of IL-1β and TNF-α (Heneka et al., 2015), which may impair hippocampal function (Intlekofer and Cotman, 2013), corroborating the idea that pro-inflammatory cytokines induce cognitive decline and memory loss in AD (Sousa et al., 2021; Almeida et al., 2022).

In animal models of AD, RE was shown to inhibit the secretion of pro-inflammatory cytokines, probably by modifying microglial activation in the hippocampus (Hashiguchi et al., 2020; Liu et al., 2020) and frontal cortex, and improving the cognitive performance of transgenic mice for AD (Liu et al., 2020). Hashiguchi et al. showed that RE restored the levels of pro-inflammatory IL-1α and IL-6 and anti-inflammatory IL-4 cytokines to control levels (Hashiguchi et al., 2020). Using a different RE protocol, Liu et al. showed that RE was able to decrease the levels of pro-inflammatory cytokines (TNF-α and IL-1β mRNA), inhibiting pro-inflammatory intracellular pathways (Liu et al., 2020). These results corroborate the results of human studies using the RE program in patients with MCI, therefore proposing the exercise as a modulator of systemic inflammation, which could explain the neuroprotective effect of the exercise (for review see Almeida et al., 2022; Huuha et al., 2022). Similar to RE, when considering aerobic exercise, studies with patients with AD have shown that, after performing an aerobic program, there was a reduction in inflammatory parameters in association with clinical improvements (Jensen et al., 2019; Li et al., 2020).

## 2. Conclusion

As shown in this mini-review, interest in RE for preventing, treating, or slowing the neuropathological conditions of AD has begun to attract researchers’ attention owing to a growing body of evidence showing the effectiveness of this type of exercise in patients with MCI and AD, and AD models in rodents. RE has been proposed as a promising strategy for reducing Aβ deposition and plaques, neurofibrillary tangles, and neuroinflammation, as well as for increasing levels of neurotrophic factors and neurogenesis, leading to improvements in memory deficits and cognitive decline. According to the WHO, RE is the recommended exercise for older people due to its effects on reducing difficulties in functional capacity, besides its benefits in muscle strengthening, better postural stability, and reduced risk of falls.

Given this scenario, RE can be proposed for patients with AD, as an alternative and adjuvant therapy, as a possible therapeutic strategy, not only to improve symptoms, but also to prevent or control the progression of neurodegeneration in AD. Considering the need to find effective strategies to decelerate the progress or even prevent AD and other types of dementia, RE seems to have preventive potential, alone or in combination with other types of exercise, thereby increasing the chance of positive results with conventional therapies and helping improve the quality of life of these patients. Moreover, insights into the effects of RE on AD may help understand the therapeutic potential of RE and the mechanisms for improving or stabilizing the disease, continuing the advances in AD therapies.

## Author contributions

DH, AP, EF, and BL wrote the first draft of the manuscript. CA, HC, EF, SO, and RA contributed to the article’s literature search. RA and BL contributed to the conception. DH, AP, EF, CA, HC, SO, RA, and BL contributed to the revision of the manuscript. All authors contributed to the article and approved the submitted version.
